# Sulfonic Acid Functionalization of Different Zeolites and Their Use as Catalysts in the Microwave-Assisted Etherification of Glycerol with *tert*-Butyl Alcohol

**DOI:** 10.3390/molecules22122206

**Published:** 2017-12-12

**Authors:** Rafael Estevez, Ivan Iglesias, Diego Luna, Felipa M. Bautista

**Affiliations:** Department of Organic Chemistry, Instituto Universitario de Investigación en Química Finay Nanoquímica (IUIQFN), University of Córdoba, Campus de Excelencia Internacional Agroalimentario (CeiA3), Marie Curie Building, E-14071 Córdoba, Spain; ivaniglesias1994@gmail.com (I.I.); qo1lumad@uco.es (D.L.)

**Keywords:** zeolite functionalization, organosilica precursor, etherification, glycerol, *tert*-butyl alcohol, microwave

## Abstract

The etherification of glycerol with *tert*-butyl alcohol in the liquid phase, over different sulfonic acid functionalized zeolites, has been studied. The reaction was carried out using microwaves as a way of heating, measured at autogenous pressure and without any solvent. Dealuminated HY and HZSM-5 zeolites by acid treatment were functionalized with two different organosilica precursors: 3-mercaptopropyltrimethoxysilane (**M**), which incorporates thiol groups, and 2-(4-chlorosulfonylphenyl)ethyltrimethoxysilane (**C**), which incorporates the sulfonic acid groups directly. The thiol groups were oxidized into sulfonic groups employing hydrogen peroxide. The textural and structural properties of the solids were studied by XRD and N_2_ adsorption–desorption isotherms, whereas the incorporation of the organosilica in the zeolites was studied by TGA and XPS. The novelty functionalization of **M** gave rise to solids with the highest acidity, and exhibited the highest yields with more substituted ethers (Y_h-GTBE_ = 13%), at 75 °C and 15 min of reaction time. In addition to the acidity, the textural properties of the zeolites played an important role in their activity; HY, with the largest size of the channels, were more active than the HZSM-5.

## 1. Introduction

The possibility of using biodiesel as a competitive alternative to fossil fuels has led to an increase in its production, especially in the last few decades. The production of biodiesel has the disadvantage that glycerol is generated as a by-product (10 wt. % of the total biodiesel produced). This fact has generated a large surplus of glycerol waste and consequently, a decrease in the glycerol price in the market [1]. Thus, the development of technologies and processes capable of converting glycerol into value-added products, is still of paramount importance.

Among the different catalytic reactions, in order to obtain fine chemicals from glycerol [2,3,4], the etherification of alcohols, specifically with *tert*-butyl alcohol (TBA), has become an interesting alternative. The main reason is that the di-*tert*-butyl glycerol ethers (DTBGs) and tri-*tert*-butyl glycerol ether (TTBG), the so-called “high ethers” (h-GTBE), can be used as excellent diesel and biodiesel additives [5]. However, the mono-*tert*-butyl glycerol ethers (MTBGs), which are also generated during the reaction, cannot be blended with diesel, making its use as an additive impossible. Thus, the reaction must be shifted towards the h-GTBE formation.

Different catalytic systems have been employed to perform this reaction, and the acid exchange resins show the most remarkable performances, concretely, the Amberlyst 15 (A-15) [6,7,8]. Nevertheless, the A-15 presents different drawbacks, such as swelling and shrinking in organic media, a lack of thermal stability, and a poor tolerance to water. Hence, the search for new heterogeneous catalysts is still challenging. In this sense, good glycerol conversion and selectivity towards desired products have been achieved employing other catalysts, such as sulfonated black carbons [9,10,11], heteropolyacids and some acid monomers supported on silica [12,13], as well as hybrid silicas and amorphous organosilica-aluminum phosphates [14,15].

**Scheme 1:**
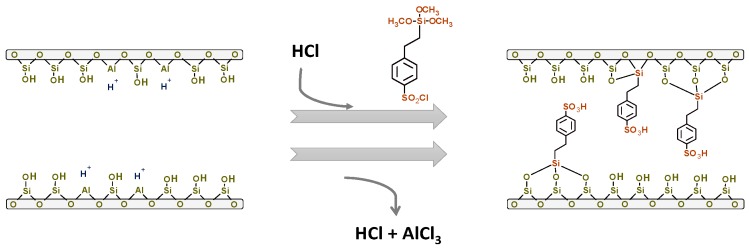
Mechanism of sulfonation of a beta zeolite with 2-(4-chlorosulfonilphenyl)ethyltrimethoxysilane (**C**) proposed by González et al. [18].

Likewise, different types of zeolites have also been employed as catalysts in this reaction, not only employing TBA as a reactant, but also isobutene (IB). Using IB as a reactant, different large-pore zeolites, such as HY and H-Beta, have been investigated, attaining a glycerol conversion around 89% on a HY zeolite, although the TTBG yield was very low [7]. The modification of a NH_4_-Y zeolite with different acid solutions gave rise to a h-GTBE yield of 49%, after 7 h and at 70 °C, on a HY zeolite washed with a citric acid solution [16]. In addition, as seen in Scheme 1, González et al. [17,18] carried out the post-sulfonation of different types of zeolites using 2-(4-chlorosulfonilphenyl)ethyltrimethoxysilane (**C**), attaining total glycerol conversion and selectivity to h-GTBE of 83% on a sulfonated beta zeolite. Regarding the employment of zeolites in the etherification of glycerol with *tert*-butyl alcohol, González et al. have reported the maximum h-GTBE yield of 27% on a fluorinated beta zeolite at 75 °C after 24 h of reaction [19].

**Scheme 2:**
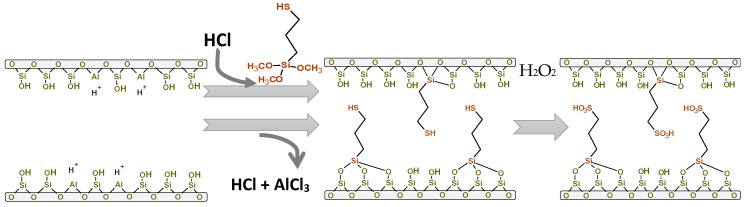
Mechanism of sulfonation of the zeolites here employed using 3-mercaptopropyltrimethoxysilane (**M**) and subsequent oxidation with H_2_O_2_.

In the present research, an original procedure for the incorporation of sulfonic groups into two commercial zeolites (HY and HZSM-5) with different SiO_2_/Al_2_O_3_ ratios and textural properties has been employed. This procedure consisted of two steps, as shown in Scheme 2. In the first step, an organosilica precursor, the 3-mercaptopropyltrimethoxysilane (**M**), was incorporated into the dealuminated zeolite and in the second one, the incorporated thiol groups were oxidized to sulfonic groups by hydrogen peroxide, at two different oxidation times, 3 and 24 h. To the best of our knowledge, there are few references about the post-sulfonation of zeolites using **M** as a grafting agent [20,21]. Likewise, for comparison purposes, the zeolites were also functionalized employing (**C**) as an organosilica precursor (Scheme 1). All the zeolites were tested in the microwave-assisted etherification of glycerol with *tert*-butyl alcohol under the same experimental conditions (75 °C, TBA/G molar ratio of 4, catalyst loading of 5 wt. % referred to initial glycerol mass and 15 min of reaction time), as those selected in our previous studies [14,15].

## 2. Results and Discussion

### 2.1. Characterization of Catalysts

**Figure 1:**
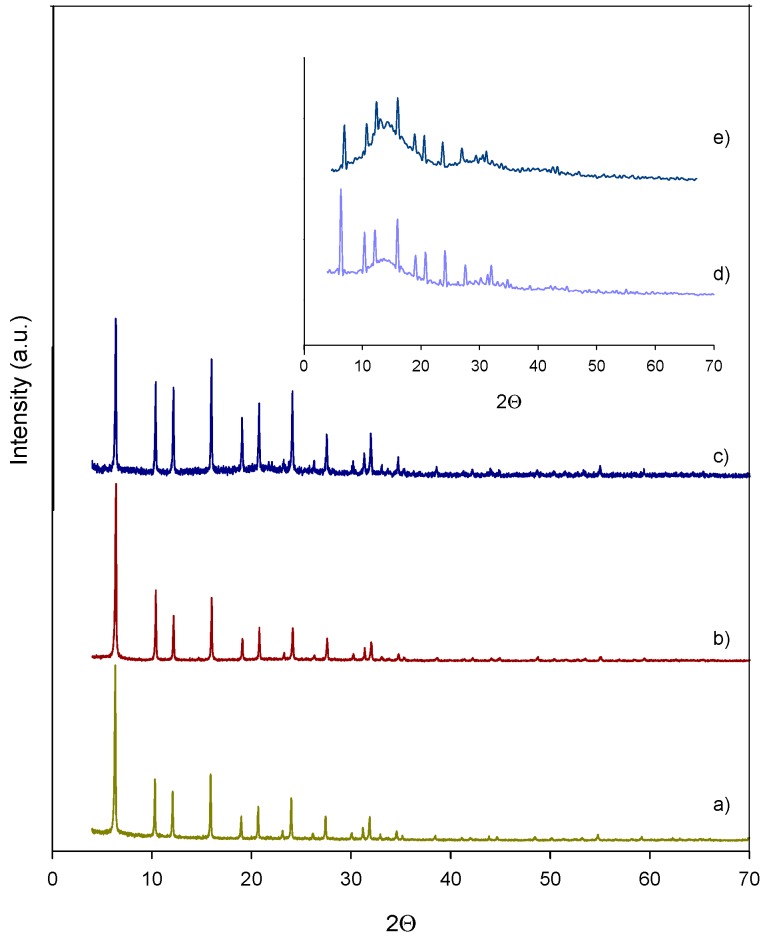
XRD patterns of (**a**) HY (**b**) C-HY (**c**) M-HY (**d**) M-HY-O(24) (**e**) M-HY-O(3).

**Figure 2:**
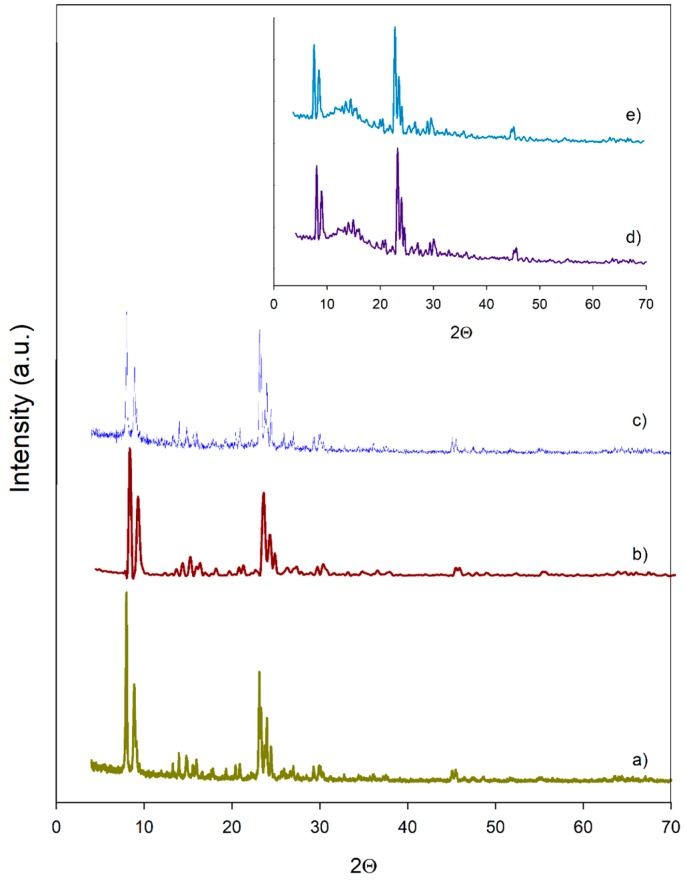
XRD patterns of (**a**) HZSM-5 (**b**) C-HZSM5 (**c**) M-HZSM-5 (**d**) M-HZSM-5-O(24) (**e**) M-HZSM-5-O(3).

The XRD patterns of the commercial and post-functionalized zeolites are shown in Figure 1 and Figure 2. As can be seen, HY maintained their cubic structure (Faujasite) after the functionalization with **C** and **M** (see Figure 1b,c), as was corroborated by the principal “hkl” indexes (111, 331 and 533). Likewise, the HZSM-5 also maintained its MFI structure after functionalization (see Figure 2b,c), according to their “hkl” indexes (101, 011, 200, 020, 501, 051). Similar results have been reported on different types of zeolites, using **C** as the organosilica precursor [17]. However, the oxidation treatment of the M-HY and M-HZSM-5 caused some decrease in the crystallinity of the zeolites (Figure 1d,e, and Figure 2d,e).

**Figure 3:**
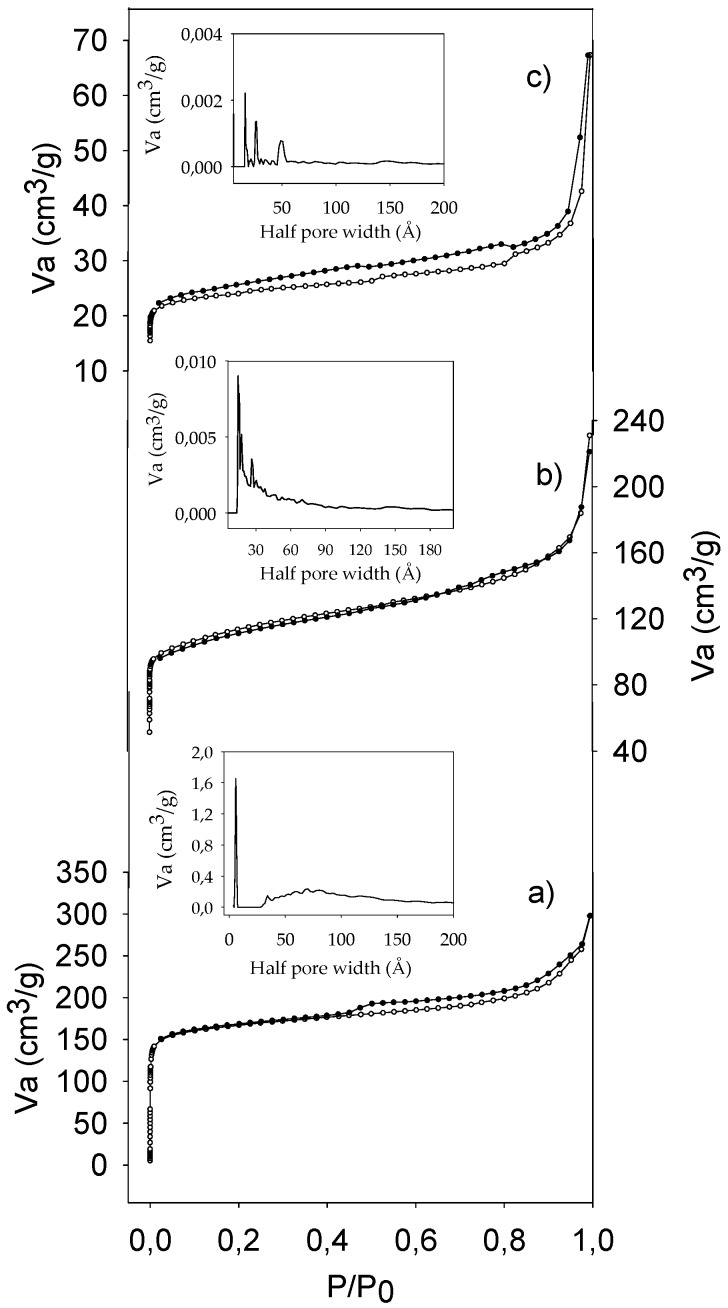
N_2_ adsorption–desorption isotherms of (**a**) C-HZSM-5 (**b**) M-HZSM-5 (**c**) M-HZSM-5-O(3).

Regarding the textural analysis, the functionalized zeolites exhibited nitrogen type I isotherms (Figure 3), while the commercial ones (not shown), corresponded to microporous solids. The textural properties of all the zeolites studied are listed in Table 1. As can be seen, the modification with **C** led to a small change in the S_BET_ of both zeolites, HY and HZSM-5, as well as an increase in the external surface (S_ext_) and a decrease in the microporous surface (S_microp_), these changes being more appreciable in the C-HY zeolite than in the C-HZSM-5 zeolite. These results could be explained taking into account the differences in the composition and size of the channels of both zeolites. The HY zeolite, with a higher amount of Al_2_O_3_ than HZSM-5 (six times higher), would undergo the dealumination process to a greater degree and hence, the number of Si–OH groups capable of interacting with the –OH groups of the organosilica precursor, would be higher, according to the mechanism shown in Scheme 1 [18]. In fact, the difficulty in the dealumination of the ZSM-5 zeolite has been previously reported [22,23]. Furthermore, the larger channel size in the HY zeolite allows a better diffusion of the precursor through its channels in comparison to the HZSM-5, implying a lower decrease in the S_microp_.

The incorporation of **M** (Scheme 2), which presents a more flexible structure and a smaller size than **C**, gave rise to a more pronounced change in the textural properties (Table 1), as was expected. In fact, a high decrease in the S_BET_ and the pore volume as well as a higher change in the pore size distribution (Figure 3) for both zeolites was observed. Furthermore, the oxidation treatment clearly had an influence on the textural properties of the M-zeolites (Table 1), leading to an increase in the S_BET_, especially after 24 h of oxidation time. A possible explanation could be the rupture of Si–O bonds of organosilica precursor, and the subsequent leaching of these precursors in the washing process after oxidation.

**Figure 4:**
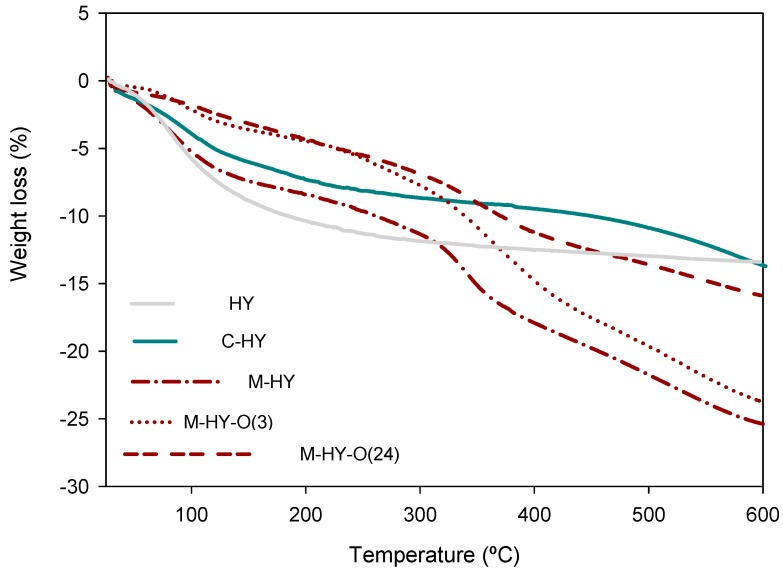
TG analysis for the HY zeolite, before and after functionalization.

The leaching of the organosulfonic groups can be corroborated by the results obtained in the thermogravimetric analysis (TGA), seen in Figure 4, given that the weight loss in the range 310–600 °C is related to the loss of organosulfonic acid groups [15,24]. Thus, whereas the M-HY and M-HY-O(3) zeolites lost 13% of weight in this range, those oxidized at 24 h lost around 9%. Furthermore, the weight loss observed in this range for the C-HY zeolite was quite low (4.5%), indicating a lower incorporation of **C** than **M** in the HY zeolite, as aforementioned.

Moreover, the weight losses observed for the HZSM-5 zeolites (commercial and modified) were similar (Figure S1), although slightly lower than those obtained on HY, mainly with the organosilica precursor **C**. This fact is once again in agreement with both the lower tendency to dealumination of this zeolite and the smaller size of their channels that the HZSM-5 exhibited, leading to a lower incorporation of the organic groups in the zeolite structure.

### 2.2. Determination of the Solid Acidity

The acidity values obtained by acid-base titration for all the solids studied are listed in Table 1. It is worth noting that the acidity of the unmodified zeolites was negligible (0.1–0.2 mmol H^+^/g). In addition, the acidity values are in agreement with the results obtained by TGA and also with the textural properties of the zeolites. In fact, the highest values of acidity were obtained on the M-HY-O(3) and the M-HZSM-5-O(3), in which the incorporation took place to a higher extent. Furthermore, a longer oxidation time led to a decrease in the acidity of the solids. Likewise, the acidity values of the modified HY were higher than those obtained on the modified HZSM-5.

**Figure 5:**
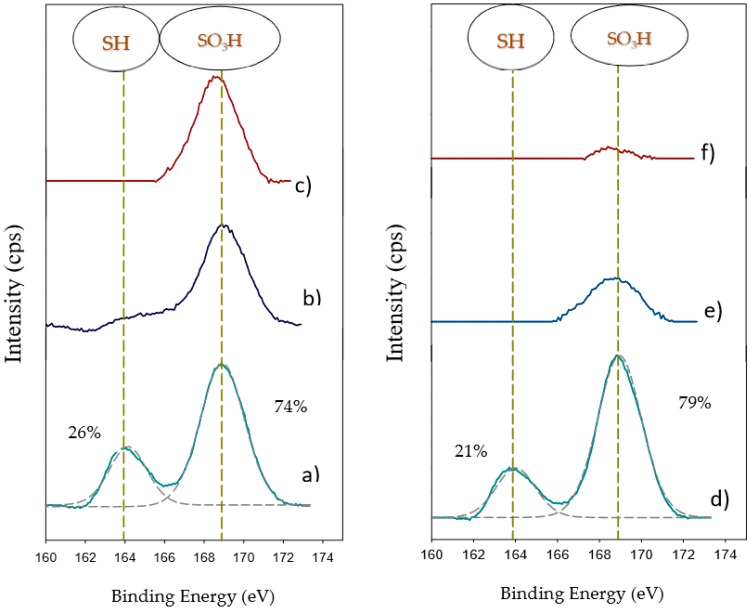
S2p XPS spectra for (**a**) M-HY-O(3) (**b**) M-HY-O(24) (**c**) C-HY (**d**) M-HZSM-5-O(3) (**e**) M-HZSM-5-O(24) (**f**) C-HZSM-5.

The transformation of the thiol groups into sulfonic acid groups was studied by XPS of sulfur (Figure 5), given that the peak around 169 eV is assigned to the sulfur in sulfonic form (-SO_3_H) [24,25], whereas the peak around 164 eV is related to the sulfur in the thiol form (-SH). In fact, the XPS spectra of the C-zeolites only exhibited the peak at 169 eV, whereas the spectra of the M-zeolites exhibited both peaks. The oxidation extent was similar for both zeolites after 3 h of oxidation time (Figure 5a,d). Furthermore, the leaching of the organo-sulfonic groups after 24 h of oxidation treatment can also be verified (Figure 5b,e). It is worth noting the good correlation between the acidity values obtained from the amount of sulfur determined by TGA and the oxidized sulfur from XPS and the acidity values obtained by acid-base titration, Table 1.

### 2.3. Microwave-Assisted Etherification of Glycerol

Table 2 shows the catalytic activity results obtained for all the catalysts in the microwave-assisted etherification of glycerol with *tert*-butanol after 15 min of reaction.

The reaction products obtained were MTBGs, DTBGs and, in some cases, low amounts of TTBG. Furthermore, small amounts of isobutene were also detected, although they were not considered for quantification because they came from TBA, which was in excess. Moreover, secondary reactions coming from glycerol were not obtained under these conditions.

**Figure 6:**
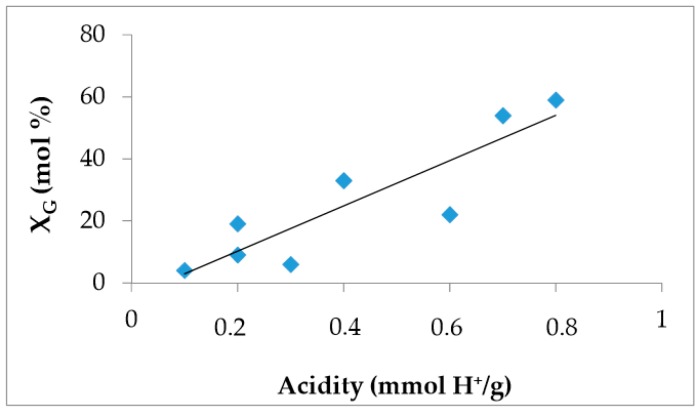
Conversion values obtained at 15 min (Table 2) vs acidity obtained by titration (Table 2).

As a general tendency, the most acidic catalysts exhibited the highest values of glycerol conversion and selectivity to h-GTBE (Table 2). In fact, a good relationship between the acidity of the solids and the glycerol conversion values was obtained (Figure 6). 

This fact is in agreement with the mechanism proposed in literature and one that we also previously corroborated employing different catalytic systems [14,15]. Thus, the reaction occurs via a fast protonation of the TBA on acid sites, giving rise to a tertiary carbocation that reacts with glycerol, generating the different reaction products.

For a better comparison among the best catalysts studied here and those reported in literature, including the commercial A-15, the Site Time Yield (STY) was calculated, as mmol of h-GTBE per mmol of H^+^ and per hour. As can be seen in Table 3, the best STY values were obtained on the modified HY zeolites, the value obtained on C-HY (174 h^−1^) being slightly higher than that obtained on M-HY-O(3) (149 h^−1^). The higher strength of the arenesulfonic acid groups in comparison with the alkylsulfonic ones would explain this behavior. Furthermore, the results obtained on the modified HY zeolites were comparable or even slightly superior to those obtained on a synthetic organosilica-aluminum phosphate (152 h^−1^) as we recently reported [15], indicating the good activity achieved on these zeolites. In addition, the catalytic activity of the C-HY, M-HY-O(3) and the M-HZSM5-O(3) clearly surpassed that obtained on the A-15, despite the fact that this A-15 exhibited more acidity than the zeolites.

This fact, which has previously taken place on other catalytic systems, such as hybrid silicas and different organosilica-aluminum phosphates, can be ascribed to the higher hydrophilicity of the synthetic catalysts in comparison to the A-15. In this solid, the only hydrophilic part of its structure are the sulfonic groups, which are susceptible to a quicker deactivation by the water generated during the reaction [14,15]. Analogously to the synthetic catalysts, the zeolites studied here exhibit a higher hydrophilic character than A-15, showing better catalytic activity.

The reusability of the zeolite, M-HY-O(3), showing the best catalytic behavior in Table 2, has been studied. With this purpose, the zeolite recovered was washed with ethanol and dried at 80 °C and afterwards, was employed again in the etherification reaction. As can be seen in Table 2, the glycerol conversion significantly decreased, although the selectivity to the different products remained practically constant. At this point, the acidity of the spent solid was measured twice, in order to rule out the leaching of the active sites. They obtained a very similar acidity value (0.7 mmol H^+^/g) to that obtained of the fresh solid (Table 1). Hence, the decrease in the conversion value could be ascribed to the adsorption of some reactants and products of the reaction on the catalyst, as this took place on some amorphous organosilica-aluminum phosphates, and also on different hybrid silicas [14,15]. In fact, after undergoing an extraction procedure with ethanol under reflux for 3 h, the zeolite exhibited a glycerol conversion of 53% with a h-GTBE yield of 10%, which was similar to those obtained on the fresh M-HY-O(3), seen in Table 2 and Table 3.

On the basis of the results obtained here, the functionalization of zeolites in order to incorporate sulfonic groups in their structure would be an interesting option to accomplish the microwave-assisted etherification of glycerol with *tert*-butyl alcohol, giving rise to high values of STY for this reaction.

## 3. Materials and Methods

### 3.1. Synthesis of Catalysts

Two commercial zeolites from Zeolyst, one ZSM-5 (CBV 3024E) and one Zeolite Y (CBV 600), were modified employing 0.8 g (4 mmol) of 3-mercaptopropyltrimethoxysilane (**M**). After drying overnight, 0.6 g of each material was subjected to oxidation with 20 mL of H_2_O_2_ at room temperature for 3 and 24 h. These solids were denoted as M-HY-O(**x**) and M-HZSM-5-O(**x**) where **x** is the oxidation time.

For the modification with **C**, 2 g of each commercial zeolite was treated with 1.4 g of **C** (4 mmol) in a 2 M HCl solution by refluxing at 40 °C for 3 h. Samples were filtered off, washed with deionized water and dried overnight at 120 °C. Sulfonic acid-functionalized zeolites were called C-HY and C-HZSM-5.

### 3.2. Characterization of Catalysts

XRD powder diffraction patterns were obtained using a Discover (Bruker, Billerica, MA, USA), diffractometer equipped with Cu Kα radiation. Finely ground samples were scanned at a speed of 2°/min (2θ = 2–70°). Textural properties were determined from the N_2_ adsorption–desorption isotherms at −196 °C, using a Quantachrome autosorb (Boynton Beach, FL, USA) iQ apparatus. Prior to measurements, all the samples were degassed at 120 °C for 12 h. The specific surface area of each solid, SBET, was determined by using the BET method at relative pressures in the range p/p0=0.05−0.3 assuming a cross-sectional area of 0.162 nm^2^ for the nitrogen molecule. The values of pore-volume and the pore-size distributions were calculated by the Density Functional Theory method (DFT). Thermogravimetric analyses (TGA) were recorded on a Setaram Setsys (Caluire, France) 12 thermal analysis stations by heating air from 30−600 °C at a rate of 10 °C min^−1^. XPS spectra were recorded with a SPECS Phoibos HAS 3500 150 MCD (Berlin, Germany) being the residual pressure in the analysis chamber 5 × 10^−9^ Pa. Accurate binding energies were determined with respect to the position of the Si 2p peak at 103.4 eV. The peaks were decomposed using a least-squares fitting routine (CasaXPS software, SPECS, Berlin, Germany) with a Gaussian–Lorentzian (70:30) using Shirley baselines. The acidity was determined using the following procedure. Aliquots of 50 mg of the material were stirred with 20 g of a 2 M NaCl solution at room temperature for 24 h. Then, the solid was filtered off, and the resulting solution titrated with a 0.025 M NaOH solution using phenolphthalein as an indicator.

### 3.3. Microwave-Assisted Etherification of Glycerol

Microwave experiments were carried out in a CEM-DISCOVER apparatus (Matthews, CA, USA) with PC control. Experiments were performed on a pressure-controlled, closed vessel under continuous stirring. The microwave method was generally power-controlled, where the reaction mixture was irradiated until the temperature selected, measured by an infra-red probe, was reached. In a typical run, the composition of the reaction mixture was 0.4 g of glycerol, TBA/G molar ratio of 4, and a constant catalyst loading of 5 wt. % (referred to as initial glycerol mass), based on previous experiments [14,15]. The total volume of the reactant mixture was 2 mL. The reaction temperature was 75 °C and after 15 min of reaction, the sample was cooled down in an ice bath, filtered off and subsequently analyzed.

Furthermore, blank experiments using either microwave or conventional heating, showed that the mixture of TBA/G did not react in the absence of a catalyst under the experimental conditions employed.

The identification of the products was carried out by GC-MS (VARIAN CP 3800, QUADRUPOLE MS 1200) (Santa Clara, CA, USA) equipped with a Supelcowax 10 capillary column, 100% ethylene glycol (30 m × 0.25 mm × 0.25 µm), according to the characterization data reported by Jamróz et al. [26]. The quantitative analysis was carried out by GC in a Hewlett Packard 5890 series II, also equipped with a Supelcowax 10 capillary column, and a FID detector, using 4-chlorotoluene as an internal standard [14,15]. Site Time Yield (STY) was calculated for all the catalysts, as mmol of h-GTBE produced, per mmol of active site (SO_3_H) and per hour.

## 4. Conclusions

Commercial zeolites (HY and HZSM-5) have been functionalized by an original procedure, employing the 3-mercaptopropyltrimethoxysilane (**M**) organosilica precursor that incorporates organothiol groups, which were subsequently oxidized using hydrogen peroxide. Another 2-(4-Chlorosulfonilphenyl)ethyltrimethoxysilane (**C**) organosilica precursor, which directly incorporates a sulfonic group in the structure was also utilized. The incorporation of the organosilica precursors on the HY zeolite was favoured due to the large size of their channels and the easier dealumination. Furthermore, the incorporation of **M** is easier than that of **C**, due to the smaller size of the molecule and its more flexible structure. The best catalytic results were obtained on the most acidic catalysts, i.e., C-HY (STY = 176 h^−1^), and also on the M-HY and M-HZSM-5 after 3 h of oxidation time (149 and 130 h^−1^, respectively), whereas longer oxidation times led to a decrease in the activity, because the leaching of the active sites took place. The highest value obtained on the C-HY zeolite can be ascribed to the higher acid strength of the arene-SO_3_H acid groups than that of the propyl-SO_3_H ones. The results obtained on these catalysts were comparable to, or even higher than, those found in literature. The reusability of the best functionalized zeolite, M-HY-O(3), has been confirmed. 

## Figures and Tables

**Table 1:** Textural properties and acidity of all the solids studied.

Catalyst	S_BET_ (m^2^/g)	S_ext_ (m^2^/g)	S_microp_ (m^2^/g)	V_P_ (cm^3^/g)	Meso (%)	Micro (%)	NaOH Consumed (mL)	Acidity (mmol H^+^/g)	Acidity (mmol H^+^/g) ^b^
HY ^a^	545	54	491	0.36	44	56	0.30	0.2	−
C-HY	597	125	472	0.27	35	65	0.80	0.4	0.3
M-HY	241	56	185	0.16	51	49	0.35	0.2	−
M-HY-O(3)	246	76	170	0.17	69	31	1.60	0.8	0.6
M-HY-O(24)	374	77	297	0.21	34	66	1.15	0.6	0.4
HZSM-5 ^a^	349	81	268	0.27	44	56	0.20	0.1	−
C-HZSM-5	337	131	206	0.29	35	65	0.35	0.2	0.1
M-HZSM-5	60	24	36	0.15	60	40	0.15	0.1	−
M-HZSM-5-O(3)	127	50	77	0.10	65	35	1.26	0.7	0.5
M-HZSM-5-O(24)	193	68	125	0.20	55	45	0.50	0.3	0.1

^a^ Data provided by Zeolyst; ^b^ Data obtained from TGA (mmol sulfur) **x** oxidized sulfure obtained from XPS (band at 169 eV).

**Table 2:** Catalytic behavior of the zeolites studied after 15 min under microwave irradiation. Reaction conditions: 5.0 wt. % of catalyst referred to initial glycerol, TBA/G ratio = 4, reaction temperature 75 °C.

Catalizador	X_G_ (%)	S_MTBG_ (%)	S_h-GTBE_ (%)
HY	9	87	13
C-HY	33	75	25
M-HY	−	−	−
M-HY-O(3)	59	78	22 (2.1) ^a^
M-HY-O(3) ^b^	30	80	20 (1.5)
M-HY-O(3) ^c^	53	81	19 (1.7)
M-HY-O(24)	22	81	19
HZSM-5	4	99	1
C-HZSM-5	19	93	7
M-HZSM-5	−	−	−
M-HZSM-5-O(3)	54	78	22 (1.5)
M-HZSM-5-O(24)	6	95	

^a^ In parenthesis, selectivity to glycerol triether (%) Spent catalyst: ^b^ after washed with ethanol; ^c^ after reflux with ethanol.

**Table 3:** Yield to h-GTBE (Y_h-GTBE_) and STY (mmol of h-GTBE produced, per mmol of H^+^ and per hour) values obtained on the solids investigated here and on several catalysts reported in the literature, after 15 min of reaction. Reaction conditions: 5.0 wt. % of catalyst referred to initial glycerol, TBA/G ratio = 4.

Catalyst	Temperature (°C)	Y_h-GTBE_ (%)	STY (h^−1^)	References
C(10)AlPO(1.5)-250	85	21	152	[15]
A-15	75	7	13	[15]
C-HY	75	8	174	This work
C-HZSM-5	75	1	58	This work
M-HY-O(3)	75	12	149	This work
M-HZSM5-O(3)	75	12	130	This work

## Supplementary Materials

The following are available online.
