# Supplementary material for: Sulfonic Acid Functionalization of Different Zeolites and Their Use as Catalysts in the Microwave-Assisted Etherification of Glycerol with tert-Butyl Alcohol
Source: Molecules. 2017 Dec 12;22(12):2206. doi: 10.3390/molecules22122206 (PMC6149981; doi:10.3390/molecules22122206)
Supplement: Supplementary file 1 [file molecules-22-02206-s001.pdf]

Supplementary Materials:

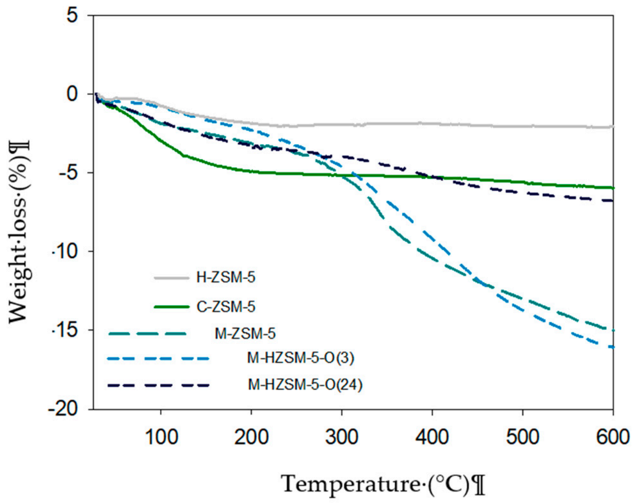

Figure S1. TG analysis for the HZSM-5 zeolite before and after functionalization.
